# Comparison between invasive radial and femoral arterial pressures and carotid tonometry in ICU patients: A physiological study

**DOI:** 10.1016/j.aicoj.2026.100068

**Published:** 2026-04-27

**Authors:** Mathieu Jozwiak, Salma Al Kahf, Emilien Umbdenstock, Jean-Louis Teboul, Denis Chemla

**Affiliations:** a Service de Médecine Intensive Réanimation, CHU de Nice, Nice, France; b UR2CA, Equipe CARRES Physiologie Cardio-Respiratoire, Université Côte d'Azur, Nice, France; c Faculté de Médecine Paris-Saclay, Université Paris-Saclay, 94270 Le Kremlin-Bicêtre, France; d INSERM UMRS 999, Hôpital Marie Lannelongue, Le Plessis-Robinson, France

**Keywords:** Cardiovascular physiology, Carotid tonometry, Intensive care unit, Femoral artery, Pulse pressure amplification, Radial artery, Systolic arterial pressure

## Abstract

**Background:**

Peripheral arterial pressure is used to guide hemodynamic management in intensive care unit (ICU) patients and for estimating various parameters of left ventricular afterload and function. Nevertheless, there is a physiological amplification of pressure from central to peripheral arteries, that remains to be documented in ICU patients. Our aim was to compare central to peripheral pressure amplification at the radial and femoral artery levels in ICU patients.

**Methods:**

In this prospective, observational and single-center study, we included consecutive spontaneously breathing patients already equipped with an arterial catheter. Carotid systolic arterial pressure obtained by carotid tonometry was considered a surrogate of aortic systolic pressure. We calculated pulse pressure amplification (PP_AMP_, difference between femoral or radial PP and carotid PP), systolic pressure amplification (SP_AMP_, difference between femoral or radial SP and carotid SP), PP ratio and SP ratio. The primary endpoint was the difference in PP_AMP_ and SP_AMP_ between the femoral and radial arteries. Secondary endpoints were the difference between peripheral and central estimates of cardiac power output (CPO), total arterial stiffness and effective arterial elastance (Ea).

**Results:**

98 patients were included: 68(69%) were men, with a mean age of 62 ± 16 years, and 42(43%) had a history of arterial hypertension. The arterial catheter was located in the radial artery in 59(60%) patients and the femoral artery in 39(40%) patients. Femoral and radial SAP and PP were higher than carotid SAP and PP (p < 0.001). The PP_AMP_ and the SP_AMP_ (12 ± 11 *vs.* 8 ± 10 mmHg p = 0.04 for both), the PP ratio (p = 0.03) and the SP ratio (p = 0.04) were higher at the radial than at the femoral artery level. Femoral and radial estimates of CPO overestimated centrally-derived CPO, with more marked overestimation at the radial than femoral artery level (9 ± 9 vs. 5 ± 7%, p = 0.04). The amount of overestimation was correlated to PP_AMP_ and SP_AMP_ (r = 0.85for both), PP ratio (r = 0.77) and SP ratio (r = 0.85) (all p < 0.001). Similar results were found for total arterial stiffness and Ea.

**Conclusions:**

The pressure amplification was lower at the femoral than radial artery level and should not be neglected in ICU patients when interpreting arterial pressures and estimating hemodynamic variables.

## Graphical abstract

**Figure:**
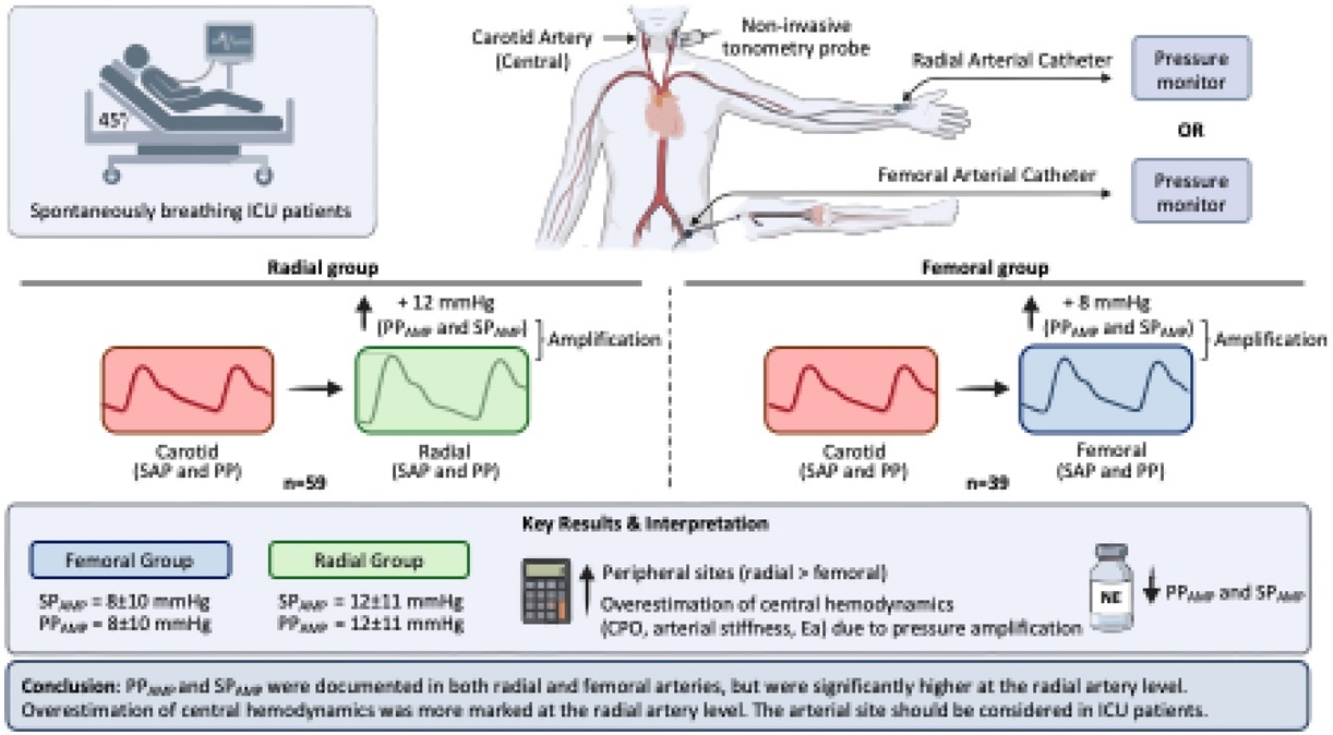

## Background

The first step in the hemodynamic monitoring of patients with shock is arterial pressure monitoring [1]. For this purpose, an arterial catheter is typically inserted into the radial or femoral artery in the most severe patients [2] and in patients not responsive to initial therapy or who require vasopressors administration [1]. The resulting peripheral arterial pressure values are then used directly to guide hemodynamic management [3]. These peripheral pressures can also serve as indirect surrogates for central aortic pressures, enabling the estimation of continuous cardiac output by pulse wave analysis [1]. In addition, they allow the calculation of several derived parameters of left ventricular (LV) afterload and function, including total arterial stiffness [4,5], effective arterial elastance (Ea) [6], and cardiac power output (CPO) [7]. The extent to which peripheral pressure can be used for such monitoring of central pressure-derived indices remains to be established.

Diastolic arterial pressure (DAP) and mean arterial pressure (MAP) are assumed to remain constant throughout the arterial tree. In contrast, systolic arterial pressure (SAP) measured at the periphery imperfectly reflects its central counterpart [8,9]. This discrepancy arises from a physiological amplification of the pulse wave, resulting in a progressive increase in SAP from the aorta to the peripheral arteries in most individuals. Because DAP remains unchanged, this phenomenon also leads to an increase in pulse pressure (PP = SAP - DAP), explaining why it is commonly referred to as pulse pressure amplification (PP_AMP_) [10]. PP_AMP_ occurs as the forward aortic pressure wave, generated by the LV ejection, travels through the arterial tree, encountering progressively stiffer and narrower vessels [10].

To date, PP_AMP_ has been mainly documented in non-critically ill patients and central pressures were indirectly estimated using a transfer function [[8], [9], [10]]. In intensive care unit (ICU) patients, PP_AMP_ has been yet poorly documented with some limitations. Firstly, central pressures were indirectly estimated with radial tonometry using a transfer function [4,5]. Secondly, previous studies focused on brachial-femoral gradient [11], on cardiac surgical patients [12,13] and considered the femoral, axillary, or subclavian artery levels as central pressures [[12], [13], [14], [15]]. Conversely, pulse wave transmission between central and peripheral arteries is generally studied between the carotid and the femoral artery [10]. PP_AMP_ has therefore never been compared between the radial and femoral artery levels with reference to a direct estimation of central pressure (carotid tonometry) in ICU patients. Such a comparison is critical for assessing the reliability of peripheral estimates of central pressures, and of CPO, total arterial stiffness and Ea.

Using simultaneous non-invasive carotid tonometry and invasive peripheral arterial pressure recordings, the primary objective of our study was therefore to compare PP_AMP_ at the radial and femoral artery levels in ICU patients. We hypothesized that PP_AMP_ would be lower at the femoral site than at the radial site, because the larger femoral artery is anatomically and functionally closer to the central aorta. The pathophysiological implications of our findings were also explored.

## Patients and methods

### Study design

This prospective and observational study was conducted in the intensive care unit of Nice teaching Hospital and was approved by the Ethics Committee of the French Intensive Care Society (agreement 12-376) and complied with the current revision of the Declaration of Helsinki. Informed consent was waived but all patients or next-of-kin were informed about the study. The study complied with the Strengthening the Reporting of Observational Studies in Epidemiology (STROBE) [16].

### Patients

Patients were included if they were already equipped with an arterial catheter and were spontaneously breathing. All transducers were attached to the patients' trunks at the phlebostatic level and zeroed against the atmospheric pressure. The transducers remained in the same place throughout the study. We included only spontaneously breathing patients to ensure a high-quality pressure signal at the carotid level. Exclusion criteria were (i) medical history of carotid occlusive diseases, (ii) heart rate >120 bpm, (iii) carotid murmur at auscultation, (iv), aortic stenosis or left ventricular outflow tract obstruction, (v) patients with non-diagnostic echocardiographic windows, defined as the inability to obtain reliable measurements to calculate the stroke volume and (vi), physician not available to perform carotid tonometry.

### Measurements and calculations

Carotid tonometry (Complior Analyse® ALAM Medical, Saint-Quentin-Fallavier, France) measurements were performed as previously described [6]. This device has been validated against invasive aortic pressure [17]. The central SAP estimated by using the tonometry-derived carotid SAP does not rely on applying a transfer function or peripheral waveforms analysis. The carotid tonometer pressure signals were calibrated from the invasive diastolic arterial pressure (DAP) and the time-averaged mean arterial pressure (MAP) obtained at the radial or femoral site, assuming unchanged DAP and MAP from the aorta to the peripheral arteries [9,10]. Carotid SAP was considered a surrogate of aortic systolic pressure and was used for calculating systolic pressure amplification from central to peripheral arteries (SP_AMP_).

The stroke volume was calculated by transpulmonary thermodilution in patients already equipped with a central venous catheter in the superior vena cava territory and a thermistor-tipped arterial catheter in the femoral artery (PiCCO-2, Getinge, Göteborg, Sweden) (n = 20) or by transthoracic echocardiography (Philips CX50, Philips Healthcare, DA Best, The Netherlands) in the other patients (n = 78), as the difference between the LV end-diastolic and end-systolic volume measured from the hand-drawn contours obtained in the apical four-chamber view : stroke volume = LV end-diastolic volume – end-systolic volume.

Carotid tonometry was performed following transpulmonary thermodilution and echocardiographic measurements. All patients were in a 45-degree semi recumbent position.

The phenomenon of pressure amplification was assessed by calculating PP_AMP_, SP_AMP_, the PP ratio and the SP ratio. PP_AMP_ (mmHg) was defined as the difference between femoral or radial PP and carotid PP and the PP ratio was defined as the ratio of femoral or radial PP to carotid PP. Similarly, SP_AMP_ (mmHg) was defined as the difference between femoral or radial SAP and carotid SAP, and the SP ratio as the ratio of femoral or radial SAP to carotid SAP.

Because it was ethically not feasible to directly evaluate the effect of pressure amplification on the accuracy of cardiac output measurements by pulse wave analysis between the femoral and radial artery level since patients should have been equipped with both femoral and radial arterial catheters for concomitant cardiac output measurements both at the femoral and radial artery level, we instead examined the influence of pressure amplification on CPO. CPO is an index of LV myocardial work, is a prognostic factor in patients with heart disease [18], heart failure [19], cardiogenic shock [20], and cardiac arrest [21], and may be incorporated into clinical decision-making algorithms for these patients. CPO (in Watts) represents the hydraulic energy delivered by the LV to the systemic circulation per unit time [22] and was calculated as: CPO = (LV mean ejection pressure × cardiac output)/451. In this study, LV mean ejection pressure was derived by integrating the systolic pressure area from the carotid pressure upstroke to the dicrotic notch and dividing by the LV ejection time. At the peripheral sites, CPO was estimated using 0.87 × femoral or radial SAP as a surrogate for LV mean ejection pressure [7].

Likewise, it is mathematically expected that the indices derived from central PP should be overestimated when peripheral PP is used, by a factor equal to the PP ratio. To illustrate this, we calculated total arterial stiffness as the ratio between central PP and stroke volume and Ea as the ratio between (0.9 × central SAP) and stroke volume [6].

### Endpoints

The primary endpoint was the difference in both PP_AMP_ and SP_AMP_ between the femoral and radial arteries. Secondary endpoints were the influence of amplification on hemodynamic on peripherally-estimated hemodynamic indices as compared to centrally-estimated ones (CPO, total arterial stiffness and Ea).

### Statistical analysis

Normal distribution of data was assessed visually using Q-Q plots. Continuous variables were expressed as mean ± standard deviation or median (interquartile range) as appropriate and categorical variables as number (percentage). Comparison between groups were performed using Student-*t*-test or Mann-Whitney test for continuous variables and Pearson’s Chi-Squared test or exact Fisher’s test for categorical variables. Correlations were assessed using Pearson's coefficient or Spearman’s rank correlation. Comparisons between central and peripheral pressures, as well as between CPO, total arterial stiffness and Ea and their estimates were performed using paired Student-test or Wilcoxon test and using the Bland-Altman method.

Physiologically speaking, both PP_AMP_ and SP_AMP_ should be higher at the radial than at the femoral artery level, since the femoral artery is considered more central than the radial artery [14,23]. Based on unpublished preliminary data from our group, we hypothesized a difference of 5 ± 8 mmHg in SP_AMP_ between the radial and femoral arteries. Therefore, it was planned to include 80 patients with an alpha risk of 5% and a power of 80%. To consider non-reliable carotid tonometry measurements in 15% of patients, we finally planned to include at least 92 patients.

All statistical analyses were performed using EasyMedStat (version 3.44; www.easymedstat.com) and a p-value <0.05 was considered statistically significant.

## Results

### Patients

**Fig. 1:**
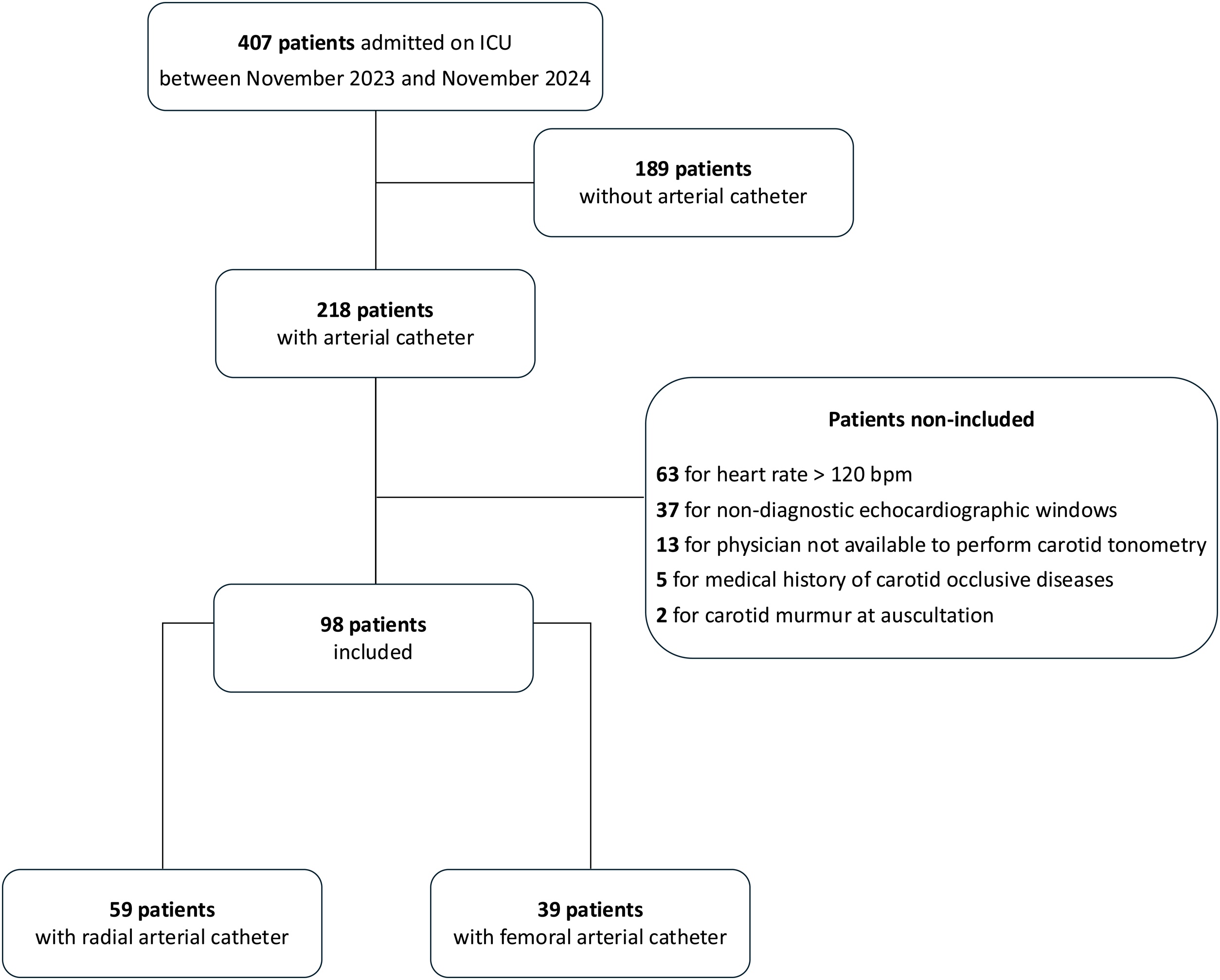
Flowchart of the study. ICU: intensive care unit.

Between November 2023 and November 2024, 218 consecutive patients were screened and 98 were included in the final analysis (Fig. 1). Patient characteristics are summarized in Table 1: 68 (69%) were men, with a mean age of 62 ± 16 years and a SAPS-2 score of 47 ± 20, 42 (43%) had a history of arterial hypertension, 5 (5%) had a history of Ilio-femoral occlusive disease and 38 (39%) were smokers. The arterial catheter was located in the radial artery in 59 (60%) patients and in the femoral artery in 39 (40%) patients (Table 1).

**Table 1:** Patient characteristics and outcomes.

	Whole population (n = 98)	Femoral catheter (n = 39)	Radial catheter (n = 59)	p value
**Patient characteristics**				
Age (years)	62 ± 16	64 ± 16	60 ± 15	0.16
Male, n (%)	68 (69)	26 (67)	42 (71)	0.63
SAPS-2	47 ± 20	56 ± 22	42 ± 17	<0.01
Body mass index (kg/m^2^)	25 ± 5	25 ± 4	25 ± 6	0.90
Arterial hypertension, n (%)	42 (43)	19 (49)	23 (39)	0.34
Smokers, n (%)	38 (39)	16 (41)	22 (37)	0.71
Diabete mellitus, n (%)	20 (20)	4 (10)	16 (27)	0.04
Dyslipidemia, n (%)	20 (20)	8 (21)	12 (20)	0.98
Ilio-femoral occlusive disease, n (%)	5 (5)	2 (5)	3 (5)	1.00
Sinusal rhythm, n (%)	87 (89)	33 (85)	54 (92)	0.34
Norepinephrine, n (%)	18 (18)	5 (13)	13 (22)	0.25
Norepinephrine dose (μg/kg/min)	0.18 (0.11–0.42)	0.20 (0.20–0.52)	0.14 (0.11–0.35)	0.37
**Outcomes**				
ICU length of stay (days)	9 ± 11	9 ± 10	10 ± 11	0.84
ICU mortality rate, n (%)	6 (6)	4 (10)	2 (3)	0.21

Variables are summarized as mean ± standard deviation, median (interquartiles) or numbers (percentages).

ICU: intensive care unit; SAPS: Simplified Acute Physiology Score.

### Carotid-femoral amplification

**Fig. 2:**
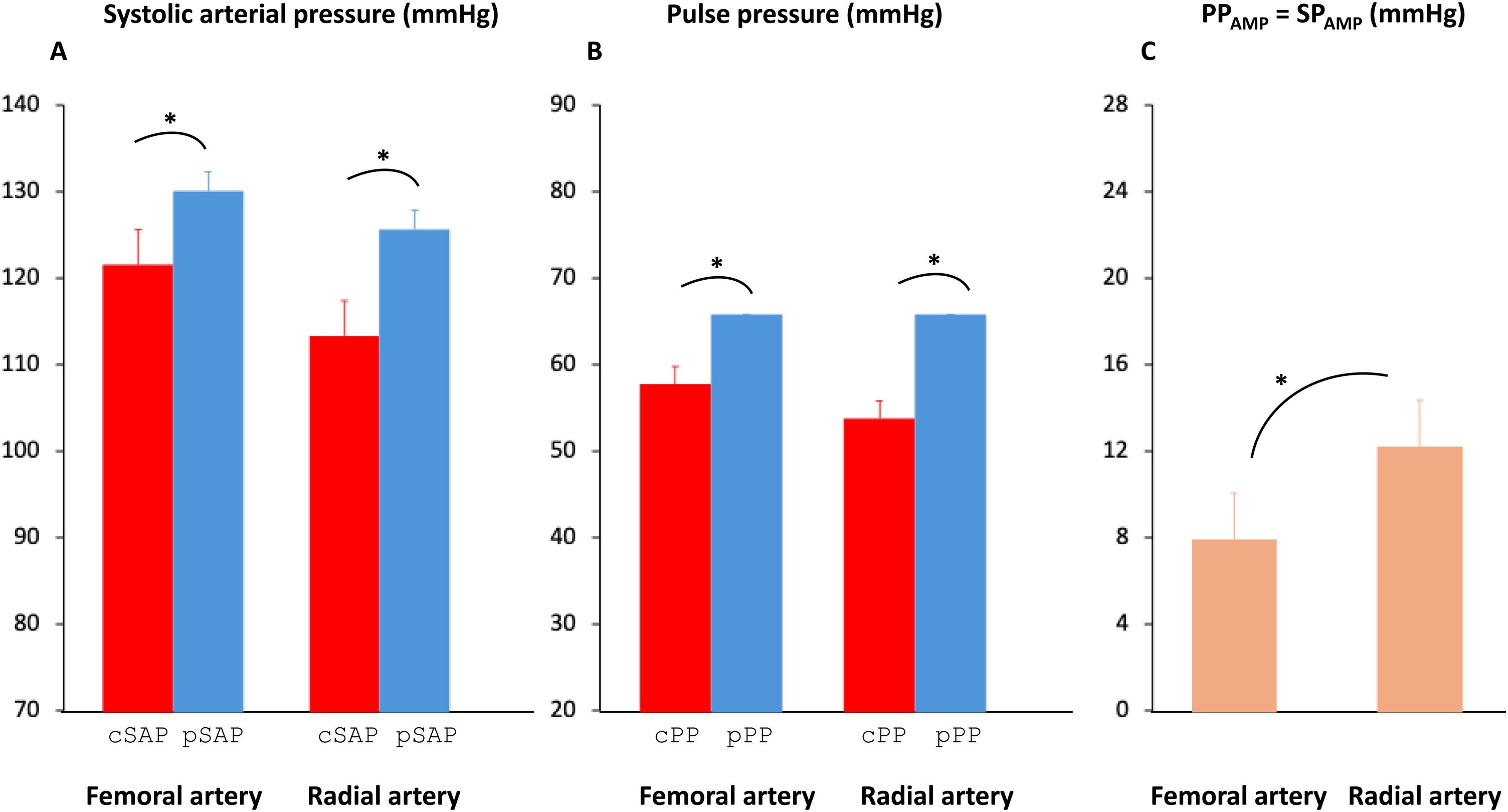
Carotid and peripheral systolic arterial pressures, pulse pressure and pulse pressure amplification (PP_AMP_) and systolic amplification (SP_AMP_) at the radial and femoral artery level. Panel A: The red bargraphs show the carotid systolic arterial pressure (cSAP) and the blue bargraphs the peripheral systolic arterial pressure (pSAP) in patients with a femoral arterial catheter (n = 39) and in patients with a radial arterial catheter (n = 59). Data are expressed as mean and standard deviation, *p < 0.05. Panel B: The red bargraphs show the carotid pulse pressure (cPP) and the blue bargraphs the peripheral pulse pressure (pPP) in patients with a femoral arterial catheter (n = 39) and in patients with a radial arterial catheter (n = 59). Data are expressed as mean and standard deviation, *p < 0.05. Panel C: The bargraphs show PP_AMP_ and SP_AMP_ in patients with a femoral arterial catheter (n = 39) and in patients with a radial arterial catheter (n = 59), assuming that the diastolic arterial pressure remains constant from the carotid to the peripheral artery level. Data are expressed as mean and standard deviation, *p < 0.05.

**Fig. 3:**
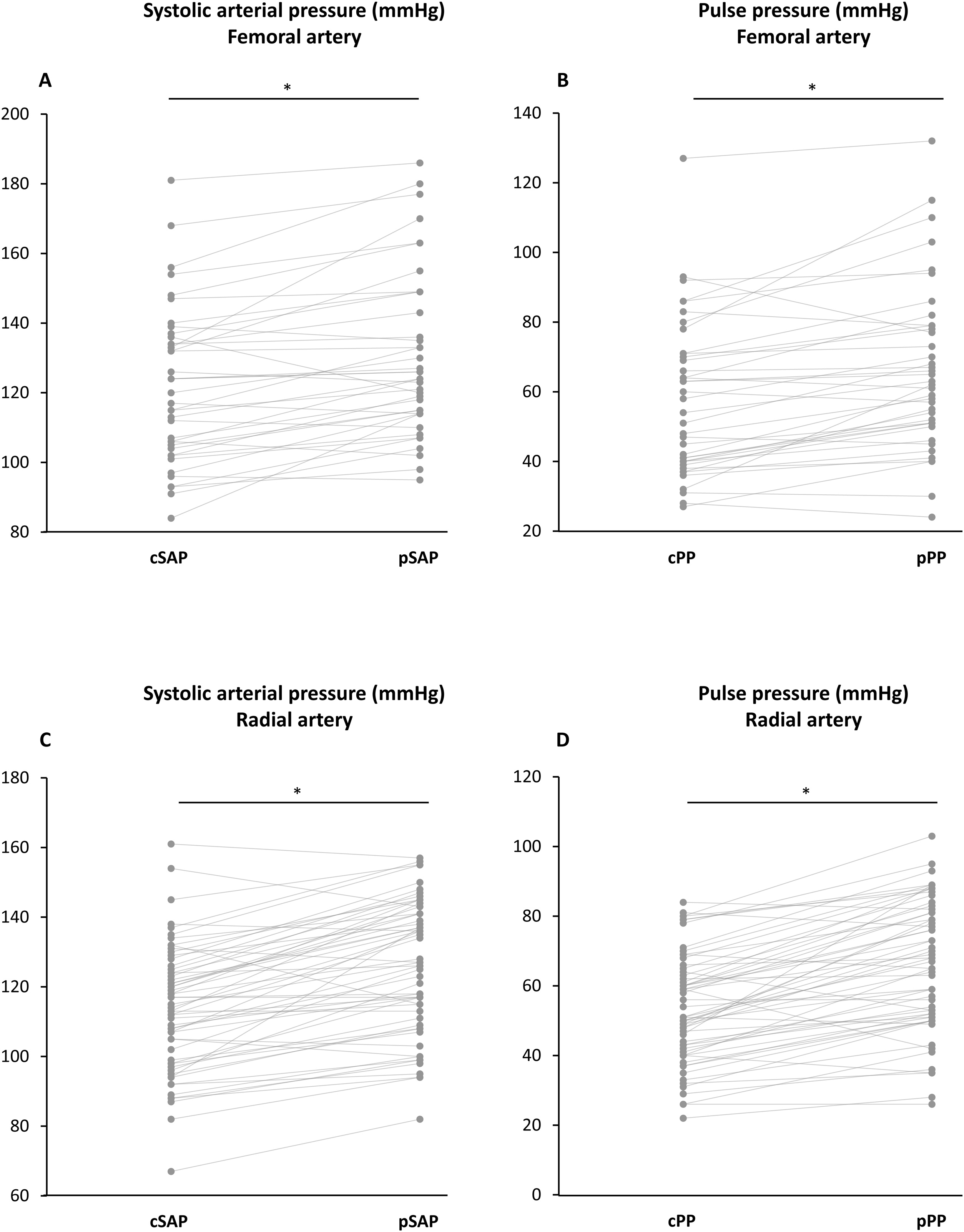
Individual values of carotid and peripheral systolic arterial pressures and pulse pressures. Panel A: Individual values of carotid systolic arterial pressure (cSAP) and peripheral systolic arterial pressure (pSAP) in patients with a femoral arterial catheter (n = 39). Each point represents a patient, *p < 0.05. Panel B: Individual values of carotid pulse pressure (cPP) and peripheral pulse pressure (pPP) in patients with a femoral arterial catheter (n = 39). Each point represents a patient, *p < 0.05. Panel C: Individual values of carotid systolic arterial pressure (cSAP) and peripheral systolic arterial pressure (pSAP) in patients with a radial arterial catheter (n = 59). Each point represents a patient, *p < 0.05. Panel D: Individual values of carotid pulse pressure (cPP) and peripheral pulse pressure (pPP) in patients with a radial arterial catheter (n = 59). Each point represents a patient, *p < 0.05.

Femoral SAP and PP were respectively higher than carotid SAP and PP (p < 0.001 for both) (Table 2, Fig. 2A and 2 B, Fig. 3A and 3 B and Fig. S1A). Both PP_AMP_ and SP_AMP_ were 8 ± 10 mmHg, as DAP was assumed to remain constant throughout the arterial tree. Conversely, the PP ratio (peripheral PP/carotid PP) was higher than the SP ratio (peripheral SAP/carotid SAP) owing to smaller denominator in the former case (1.17 ± 0.21 vs. 1.07 ± 0.09, p < 0.001) (Table 2).

**Table 2:** Central and peripheral pressures.

	Femoral catheter (n = 39)	Radial catheter (n = 59)	p value
Diastolic arterial pressure (mmHg)	64 ± 13	60 ± 11	0.09
Mean arterial pressure (mmHg)	87 ± 13	82 ± 12	0.06
Peripheral systolic arterial pressure (mmHg)	130 ± 24	126 ± 19	0.32
Carotid systolic arterial pressure (mmHg)	122 ± 22	114 ± 18	0.06
SP_AMP_ (mmHg)	8 ± 10	12 ± 11	0.04
Systolic pressure ratio	1.07 ± 0.09	1.11 ± 0.09	0.04
Peripheral pulse pressure (mmHg)	66 ± 24	66 ± 18	0.96
Carotid pulse pressure (mmHg)	58 ± 22	54 ± 15	0.32
PP_AMP_ (mmHg)	8 ± 10	12 ± 11	0.04
Pulse pressure ratio	1.17 ± 0.21	1.25 ± 0.22	0.03
Heart rate (bpm)	93 ± 16	86 ± 17	0.06

Variables are summarized as mean ± standard deviation. SP_AMP_: systolic arterial pressure amplification, PP_AMP_: pulse pressure amplification. Diastolic and mean arterial pressures are assumed to remain constant throughout the arterial tree. Therefore, the absolute value of PP_AMP_ was equal to that of SP_AMP,_ but the relative value differed, as illustrated by the different value of systolic pressure ratio and pulse pressure ratio.

### Carotid-radial amplification

Radial SAP and PP were respectively higher than carotid SAP and PP (p < 0.001 for both) (Table 2, Fig. 2A and 2 B, Fig. 3C and 3 D and Fig. S1B). Both PP_AMP_ and SP_AMP_ were 12 ± 11 mmHg. The PP ratio was higher than the SP ratio (1.25 ± 0.22 *vs.* 1.11 ± 0.09, p < 0.001) (Table 2).

The PP_AMP_ and the SP_AMP_ (p = 0.04 for both), the PP ratio (p = 0.03) and the SP ratio (p = 0.04) were higher at the radial artery level than at the femoral artery level (Table 2, Fig. 2C).

### Influence of norepinephrine on pressure amplification

Overall, 18 (18%) patients received norepinephrine at the time of hemodynamic measurements with a median dose of 0.18 (0.11–0.42) μg/kg/min (Table 1). Irrespective of the location of the arterial catheter, both PP_AMP_ and SP_AMP_ (6 ± 13 *vs.* 12 ± 10 mmHg, p = 0.01), the PP ratio (1.12 ± 0.25 *vs.* 1.24 ± 0.20, p = 0.01) and the SP ratio (1.06 ± 0.13 *vs.* 1.11 ± 0.09, p = 0.02) were lower in patients receiving norepinephrine than in patients who did not.

### Peripheral estimates of CPO

**Fig. 4:**
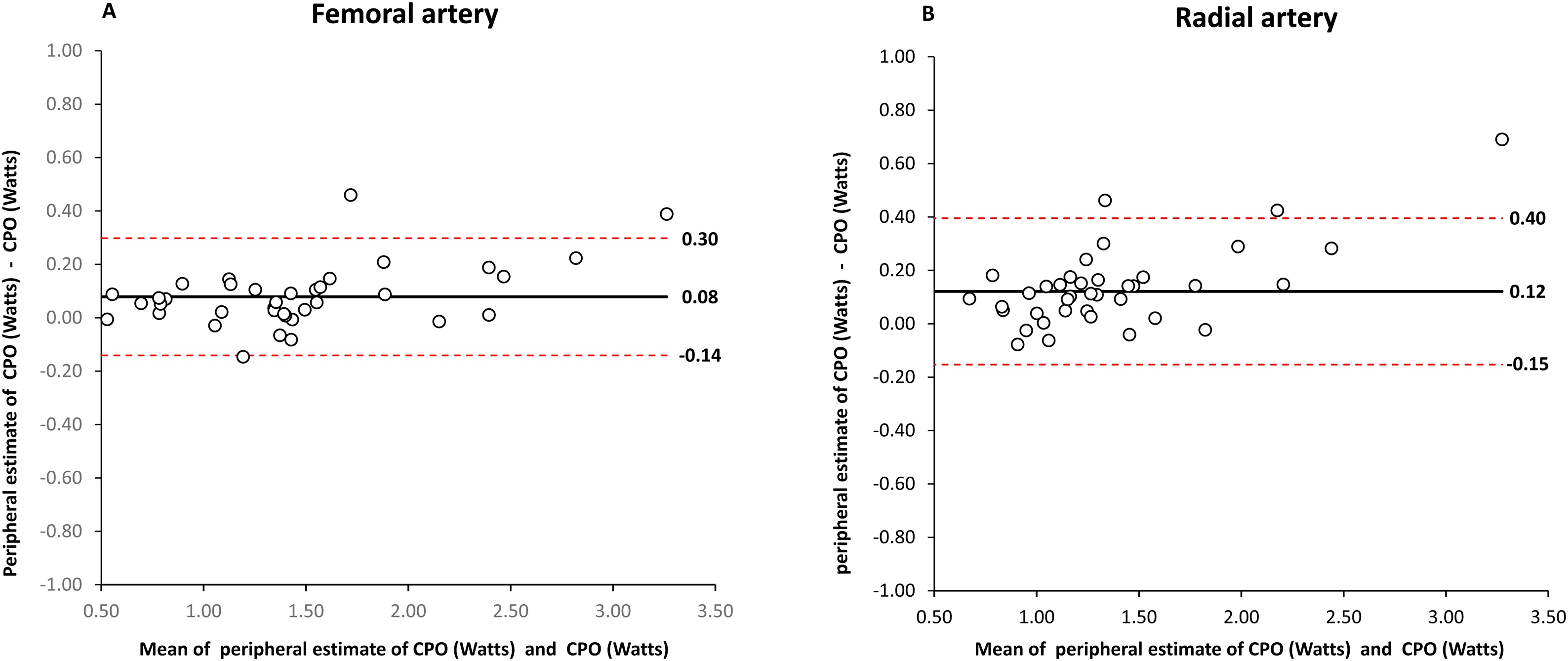
Comparison of cardiac power output (CPO) estimates and CPO. Panel A: Bland-Altman analysis between femoral estimate of CPO and CPO in patients with a femoral arterial catheter (n = 39). The solid line represents the mean bias. Dotted lines represent the limits of agreement (mean ± 1.96 standard deviation). Panel B: Bland-Altman analysis between radial estimate of CPO and CPO in patients with a radial arterial catheter (n = 59). The solid line represents the mean bias. Dotted lines represent the limits of agreement (mean ± 1.96 standard deviation).

In the whole population (n = 98), the reference CPO derived at the carotid artery level was 1.24 ± 0.55 watts. Estimates of CPO derived at both the femoral and radial levels overestimated CPO, with a more marked overestimation at the radial than femoral artery level (9 ± 9 *vs.* 5 ± 7%, p = 0.04) (Table 3, Fig. 4). The amount of overestimation was correlated to PP_AMP_ and SP_AMP_ (r = 0.85, p < 0.001 for both), PP ratio (r = 0.77, p < 0.001) and SP ratio (r = 0.85, p < 0.001).

**Table 3:** Energetic and arterial load indices.

	Femoral catheter (n = 39)	Radial catheter (n = 59)
Cardiac power output (CPO, Watts)	1.36 ± 0.63	1.17 ± 0.48
Peripheral CPO estimate (Watts)	1.44 ± 0.68*	1.29 ± 0.56*
Arterial stiffness (TAS, mmHg/mL)	1.15 ± 0.77	0.97 ± 0.44
Peripheral TAS estimate (mmHg/mL)	1.38 ± 1.05*	1.20 ± 0.56*
Arterial elastance (Ea, mmHg/mL)	2.21 ± 1.22	1.88 ± 0.80
Peripheral Ea estimate (mmHg/mL)	2.42 ± 1.49*	2.08 ± 0.90*

Variables are summarized as mean ± standard deviation.

* p < 0.05 between peripheral estimate and their central counterpart.

### Peripheral estimates of total arterial stiffness and Ea

In the whole population (n = 98), total arterial stiffness was 1.04 ± 0.59 mmHg/mL and Ea was 2.01 ± 0.99 mmHg/mL. Both femoral and radial estimates overestimated total arterial stiffness and Ea (Table 3). For stiffness, the overestimation was more marked at the radial than femoral artery level (18 ± 16 *vs.* 12 ± 14%, p = 0.03) and the amount of overestimation was correlated to PP_AMP_ and SP_AMP_ (r = 0.92, p < 0.001 for both), PP ratio (r = 0.99, p < 0.001) and SP ratio (r = 0.96, p < 0.001). For Ea, the overestimation did not differ between the radial and the femoral artery level (9 ± 8 *vs.* 6 ± 7%, p = 0.05), while the amount of overestimation was correlated to PP_AMP_ and SP_AMP_ (r = 0.98, p < 0.001 for both), PP ratio (r = 0.96, p < 0.001) and SP ratio (r = 0.99, p < 0.001).

## Discussion

In this cohort of ICU patients with spontaneous breathing, we used carotid tonometry to non-invasively estimate central aortic pressure and found that both femoral and radial SAP were higher than carotid SAP. As DAP was assumed to remain constant throughout the arterial tree, this resulted in higher peripheral than central PP. This pressure amplification explained why estimates of LV function and afterload indices calculated from peripheral pressure measurements overestimated the corresponding values derived from central pressures. The magnitude of this overestimation of CPO, arterial stiffness and Ea, was correlated with the degree of pressure amplification. Finally, we also found that PP_AMP_ and SP_AMP_ were lower at the femoral artery level than at the radial artery level, and that norepinephrine administration was associated with lower values of PP_AMP_ and SP_AMP_.

We found that both femoral and radial SAP were higher than carotid SAP. Our results are in line with those of Kroeker et al, who found in 12 healthy individuals at rest, that radial and femoral SAP exceeded central SP [8]. In ICU hemodynamically stable patients, it has also been shown that radial [4] and femoral [5] SAP exceeded central SP estimated using radial tonometry and a transfer function. Our results and others [[4], [5], [6], [7], [8], [9],^24^] confirm that when the pressure wave travels from the aorta to the periphery, both SAP and PP increase. This phenomenon is related to the physiological amplification of the pulse wave generated by the LV ejection and explained by the narrowing of arterial caliber and the increased arterial stiffness from the aorta to peripheral arteries [10]. Importantly, we found that PP_AMP_ and SP_AMP_ were lower at the femoral artery than at the radial artery level. This may be explained in part by the fact that the femoral artery is a more central artery than the radial artery given its more central location (closer to the aorta), its elastic wall structure and its large diameter [14,23].

We also found that norepinephrine administration was associated with lower values of PP_AMP_ and SP_AMP_. Norepinephrine exerts arterial vasoconstriction, which is known to reduce pressure amplification. Indeed, in this case, the reflected pressure waves return faster and thus earlier to the aorta. In patients with stiff arteries, the aortic backward pressure wave may peak during systole and not during diastole, as it would be in young and healthy individuals having more elastic arteries. At the central level, this results in a systolic peak that markedly exceeds the maximum pressure of the forward wave generated by LV ejection, leading thus to a reduced difference between peripheral (the result of the amplification of the forward wave) and central SP [[8], [9], [10]]. Conversely, the administration of vasodilators was associated with more distal wave reflection for a given peripheral blood pressure and aortic stiffness [25]. The decrease in pulse wave velocity associated with vasodilation is probably nitric oxide-mediated, as illustrated by the increase in pulse wave velocity and thus PP_AMP_ in healthy men receiving an inhibitor of nitric oxide synthase [26]. In patients receiving norepinephrine, peripheral SAP may therefore be a more reliable estimate of LV afterload due to moderate or absent pressure amplification because of increased arterial stiffness than in patients who do not receive norepinephrine. Nevertheless, it cannot be excluded that the lower values of PP_AMP_ and SP_AMP_ with norepinephrine could have different meanings depending on patient severity. While norepinephrine may induce loss of pressure amplification in less severe patients, it cannot be excluded that it may restore this phenomenon in the most severe vasodilatory shock states [27].

Reliable tools for assessment of cardiac output and cardiac function are crucial for guiding the ICU patient’s therapy. We showed that CPO calculated from peripheral arterial pressures overestimated CPO, with a more marked overestimation for the radial than the femoral CPO. This overestimation was strongly related to the phenomenon of pressure amplification, evidenced by the strong correlations between the amount of overestimation and PP_AMP,_ SP_AMP_, PP ratio and SP ratio. Similarly, both femoral and radial arterial stiffness and Ea estimates overestimated stiffness and Ea, illustrating that indices derived from central PP will be overestimated when peripheral PP is used in their calculation. The amount of overestimation was also related to PP_AMP_.

These results suggest that the phenomenon of pressure amplification should be considered to estimate with better accuracy LV afterload, LV ventricular-arterial coupling and LV myocardial work, especially in patients with marked pressure amplification and with an arterial catheter placed in a radial artery. A simple way to calculate PP_AMP_ and SP_AMP_ at the bedside without measuring carotid SAP would be to estimate central SAP from peripheral arterial pressures. To this end, a formula has been proposed to reliably estimate central SAP from peripheral MAP and DAP (DCBP = MAP^2^/DAP) and validated against invasive pressure measurements in ICU patients [28] and non-invasive pressure measurements in non-critically ill patients [29]. Further studies are needed to confirm the clinical relevance and potential utility of this novel central pressure estimate in ICU patients.

The first clinical implication of our study is that femoral arterial catheter should be preferred over radial arterial catheter to limit the confounding effects of SP_AMP_, especially in the subgroup of individuals more likely to exhibit high SP_AMP_ values, in order to obtain a more accurate estimation of SAP and a more reliable hemodynamic monitoring. The second clinical implication concerns the influence of pressure amplification on measurement of hemodynamic indices of LV function and afterload (CPO, arterial stiffness and Ea). Even though it was not possible to directly assess the influence of SP_AMP_ on cardiac output measurement by pulse wave analysis, our results suggest that SP_AMP_ could be incorporated into the proprietary algorithms of the pulse wave analysis-cardiac output monitors. This would allow to overcome the influence of SP_AMP_ and to improve the reliability of cardiac output measurement, especially in patients with the most marked pressure amplification, namely in young and vasodilated patients and in patients with a radial arterial catheter.

Our study has some limitations. Firstly, our results strictly apply to patients free of outflow tract obstruction and aortic stenosis. Secondly, it was not possible to directly investigate the influence of pressure amplification on cardiac output measurement, since no patients had both radial and femoral arterial catheters for obvious ethical reasons. It cannot therefore be excluded that potential differences we found between the femoral and radial artery level may be partly related to potential baseline heterogeneity between groups and not only related to the site of the arterial catheter. Thirdly, stroke volume measurements necessary for CPO calculation were performed from the apical four-chamber view and not from biplane measurements. Nevertheless, since both CPO and peripheral CPO estimates are calculated using the same stroke volume, any potential systematic bias introduced by the monoplane method would affect both values equally and therefore not introduce any bias. Fourthly, our results depend on the assumption that both DAP and MAP remained constant throughout the arterial tree [9,10]. In patients receiving high doses of vasopressors, MAP and DAP may slightly vary throughout the arterial tree due to impaired pressure transmission [14,30]. Nevertheless, only 18% of patients received norepinephrine in our population with a median dose of 0.18 (0.11–0.42) μg/kg/min, which remains in a range limiting the potential effect of impaired pressure transmission to peripheral arteries. Fifthly, given the limited sample size, it was not possible to assess the impact of age, arterial hypertension and diabetes mellitus on pressure amplification, while these factors are known to affect arterial stiffness. Sixthly, as carotid tonometry cannot be performed in mechanically ventilated patients due to the alteration of the pressure signal induced by the ventilator, some patients were included late in their ICU stay, which explains the low proportion of patients receiving norepinephrine but equipped with an arterial catheter. Finally, we did not assess the influence of pressure amplification on patient outcomes, as this was a physiological study.

## Conclusion

Both femoral and radial SAP were higher than carotid SAP, resulting in higher peripheral than central PP. The pressure amplification was lower at the femoral artery level than at the radial artery level, confirming that the femoral artery is a more central artery than the radial artery. The pressure amplification is a physiological phenomenon that should not be neglected in ICU patients when interpreting arterial pressures and estimating hemodynamic variables, especially in patients at risk of marked pressure amplification, when a radial arterial catheter is used to monitor arterial pressure.

## Author contributions

MJ, JLT and DC conceived and designed the study. MJ, SAK and EU collected data. MJ, SAK, EU, JLT and DC analyzed and interpreted the data. MJ and DC drafted the first version of the manuscript report. All authors contributed drafting the manuscript and approved the final version of the manuscript.

## Ethics approval and consent to participate

The study was approved by the was approved by the Ethics Committee of the French Intensive Care Society (agreement 12-376) and complied with the current revision of the Declaration of Helsinki.

## Consent for publication

Not applicable.

## Funding

No funding to declare.

## Data availability

The datasets used and/or analyzed during the current study are available from the corresponding author on reasonable request.

## Declaration of competing interest

MJ and DC received travel grants from ALAM Medical for scientific meeting, outside the submitted work. MJ is a member of the Editorial Board of Annals of Intensive Care. JLT is the Editor-in-Chief of Annals of Intensive Care. The other authors have no conflict of interest to declare.
