# Supplementary material for: Comparison between invasive radial and femoral arterial pressures and carotid tonometry in ICU patients: A physiological study
Source: Ann Intensive Care. 2026 Apr 27;16:100068. doi: 10.1016/j.aicoj.2026.100068 (PMC13137008; doi:10.1016/j.aicoj.2026.100068)
Supplement: Supplementary file 1 [file mmc1.docx]

**Comparison between invasive radial and femoral arterial pressures and carotid tonometry in ICU patients: a physiological study**

Mathieu JOZWIAK, MD PhD^1,2^ ; Salma AL KAHF, MD^1^ ; Emilien UMBDENSTOCK, MD^1^ ; Jean-Louis TEBOUL, MD PhD^3^ ; Denis CHEMLA, MD PhD^3,4^

^1^ Service de Médecine Intensive Réanimation, CHU de Nice, Nice, France

^2^ UR2CA, Equipe CARRES Physiologie Cardio-Respiratoire, Université Côte d'Azur, Nice, France

^3^ Faculté de Médecine Paris‑Saclay, Université Paris-Saclay, 94270 Le Kremlin‑Bicêtre, France

^4^ INSERM UMRS 999, Hôpital Marie Lannelongue, Le Plessis-Robinson, France

***Corresponding author***

Mathieu JOZWIAK, MD PhD

Service de Médecine Intensive Réanimation, CHU de Nice

151 route Saint Antoine de Ginestière

06200 Nice, France

[jozwiak.m@chu-nice.fr](mailto:jozwiak.m@chu-nice.fr)

***Word count :*** 3416 words

**Supplemental figures legend**

**Figure S1 : Comparison of carotid and peripheral systolic arterial pressures.**

Panel A: Bland-Altman analysis between carotid systolic arterial pressure (cSAP) and peripheral systolic arterial pressure (pSAP) in patients with a femoral arterial catheter (n=39). The solid line represents the mean bias. Dotted lines represent the limits of agreement (mean ± 1.96 standard deviation).

Panel B: Bland-Altman analysis between carotid systolic arterial pressure (cSAP) and peripheral systolic arterial pressure (pSAP) in patients with a radial arterial catheter (n=59). The solid line represents the mean bias. Dotted lines represent the limits of agreement (mean ± 1.96 standard deviation).

**Figure S1**
